# Pressurized brines in continental Antarctica as a possible analogue of Mars

**DOI:** 10.1038/srep33158

**Published:** 2016-09-12

**Authors:** Emanuele Forte, Michele Dalle Fratte, Maurizio Azzaro, Mauro Guglielmin

**Affiliations:** 1 Department of Mathematics and Geosciences, University of Trieste, via Weiss, 2 - 34128, Trieste, Italy; 2 Department of Theoretical and Applied Sciences, Insubria University, Via Dunant, 3, Varese, Italy; 3 CNR, Institute of Marine and Coastal Environment, Spianata San Raineri, 86 - 98122, Messina, Italy

## Abstract

Interest in brines in extreme and cold environments has recently increased after they have been found on Mars. Those brines can be potential new subsurface habitats for peculiar ecosystems. In the McMurdo Dry Valleys of the Antarctic, the best analogue for Mars conditions, only a few cases of brines have been identified in some perennially frozen lakes and in one case in an underground aquifer. Here, we present the occurrence of pressurized brines in a shallow perennially ice-covered lake south of 70°S in an ice-free area of Victoria Land, Antarctica. For the first time, we also imaged, by means of ground penetrating radar data, the existence of a pingo-like-feature (PLF) formed by the extrusion of brines, which has also been confirmed by borehole evidence. Those brines are fed by an underground talik external to the lake basin, enhancing the possibility of unexploited ecosystems that could find an analogue in Martian environments.

Interest in brines in extreme and cold environments has increased after they have been found on Mars^1^,^2^. Continental Antarctica and the McMurdo Dry Valleys in particular have always been considered as the best analogues of Mars because both have cold and dry climates and because they contain a suite of landforms that closely resemble those occurring on the Martian surface. Mars and the McMurdo Dry Valleys are both characterized by the slow soil formation of salts (i.e., Ca-carbonate, Ca-sulphate, and Mg-sulphate) and brines that are very important in soil development^3^,^4^. Moreover, the paucity of liquid water on the surface and the importance of sublimation in the hydrologic cycle of the McMurdo Dry Valleys is another strong analogue with Mars^3^,^4^,^5^. Among the several landforms that occur both on Mars and on Earth, frost mounds and pingos have already been highlighted^6^, although in some cases different processes should be responsible for their origins on Mars and on the Earth^6^. Nevertheless, knowledge of the brines occurring underground in the ice-free areas of continental Antarctica^7^,^8^,^9^, below ice-sealed Antarctic lakes^10^,^11^ or below the subglacial lakes of Antarctica^12^ is very limited. The habitability of the ecosystems represented by those brines^7^,^13^,^14^ is even less known. To our knowledge, only in the case of the glacial outflow known as Blood Falls, which is located at the front of the Taylor glacier^7^, the brines seem to be recharged by a sub-permafrost talik.

Lake sediments are potential palaeoclimate archives, and in Antarctica they have mainly been studied above 70°S, where the onset of the sedimentation is generally recorded to have occurred after the Last Glacial Maximum (LGM), as documented in maritime Antarctica between 28 and 21.1 kyr^15^,^16^ in Marguerite Bay, the Antarctic Peninsula or even later in the Holocene between 7.5 and 4.6 kyr^17^,^18^,^19^,^20^ in the South Shetland or on James Ross Island. An important exception is represented by the Larsemann Hills, East Antarctica, where Cromer *et al*.^21^ showed a lacustrine record that began at approximately 130 kyr BP.

South of 70°S, fresh water lakes are extremely rare and are mainly concentrated in coastal areas such as the case of Amery Oasis, where sedimentation started at approximately 12 cal kyr BP, and a core documented clear warming event at approximately 8.5 cal kyr BP^22^.

Among perennially ice covered lakes, only the large lakes of the McMurdo Dry Valleys have been extensively studied. In the Dry Valleys during the mid to late Holocene, relevant drawdowns and even complete desiccation events have been recorded, for example in the Taylor Valley^23^,^24^ and Wright Valley^25^,^26^. The case of Vida Lake in the Victoria Valley is slightly different because the ice cover is at least partially grounded^10^, and a record of changes in the accumulation and ablation of ice related to climatic changes has been preserved since 8.6 kyr BP^27^.

The paper aims to I) show the first observed case of a small perennially frozen lake south of 70°S (74°S) in which brines were identified through ground penetrating radar (GPR) investigations and sampled by successive coring, II) present the first case of a pingo-like-feature (PLF) formed by brines coming from an open talik system, and III) contribute to the understanding of the palaeoclimatic significance of the lacustrine sediments found in the lake that started to accumulate not prior to 12.4 cal kyr BP. The first two aims could contribute to a better understanding of the possible analogies between this part of continental Antarctica and the Martian surface.

The Tarn Flat area is the largest ice-free area (ca. 11 × 9 km) north to the McMurdo Dry Valley in Victoria Land (Antarctica) and is not far from the Italian Antarctic Research Station (Mario Zucchelli Station – MZS; Fig. 1a).

This ice-free area is characterized by Late Wisconsin glacial deposits, with some spectacular eskers at lower altitudes, and by outcropping Ordovician granites overlaid by sparse erratic boulders^28^,^29^.

The climate is cold and dry with a Mean Annual Air Temperature (MAAT) of approximately −14 °C (considering the last 20 years) while precipitation, which is always in the form of snow, ranges between 100 and 200 mm/yr^30^,^31^. During summers, the mean seasonal temperature is less than −2 °C^32^ and in just a few days can reach or even exceed + 4 °C.

The study area presents a very large number of lakes and ponds (64) that have dimensions from a few tens to several hundreds of metres (Fig. 1b,c). Generally, the lakes located at lower altitudes are partially or completed melted during the summer, whereas those at higher elevations are perennially frozen or only partially melted at their margins during warmer summers.

Guglielmin *et al*.^33^ in their survey of 1999 found that only 2 of the 64 lakes of the region (marked in yellow on Fig. 1b) were characterized by the occurrence of a frost mound, namely a perennial or semi-permanent debris-covered mound, which may be related to the winter freezing of water injected into the lake under hydraulic pressure. During our 2014 survey, we found a third lake with a frost mound on the surface (labelled as Lake-1 in Fig. 1b).

Lake-1 is 280 m long and 100 m wide and is elongated in the East-West direction (Fig. 1d). The perimeter of this lake is equal to 720 m, and the lake has an area of approximately 30000 m^2^. The frost mound is located in the central part of the lake (Figs 1e and 2), reaches a maximum height of 45 cm and extends within an area of approximately 500 m^2^. For comparison, another lake of similar size located approximately 300 m from Lake-1 (labelled as Lake-2 in Fig. 1b) and without any frost mound was further surveyed.

## Results

### GPR data

Eleven Ground Penetrating Radar (GPR) profiles using combined 500 MHz and 1.6 GHz antennas (Fig. 2), with a total length of more than 2 km, were gathered in the Antarctic summer of 2014/15.

Lake-1 is scoured in granitic bedrock. It is not symmetric and has two troughs, one of which is deeper, has a bedrock depth that exceeds 7 m (Figs 3 and 4), is oriented NNW-SSE and is located in the middle of the lake, whereas the other is shallower, oriented NW-SE and is placed in the northern part of the lake (Fig. 4a).

**Figure 1:**
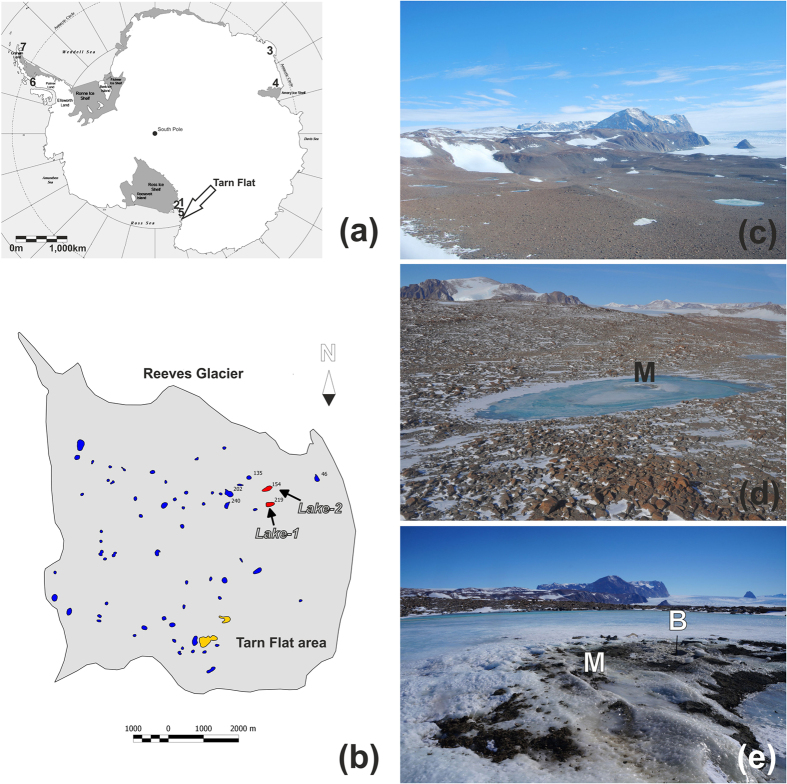
Location maps. (**a**) Position of the study area in the Antarctica obtained by the open source Generic Mapping Tools - GMT, release: GMT 5.1.2 (http://gmt.soest.hawaii.edu/projects/gmt) and edited using the CorelDRAW graphic suite, release 6X (http://www.corel.com). The numbers from 1 to 7 highlight the approximated locations of the sites cited in the text: 1) McMurdo Dry Valleys; 2) Taylor Glacier; 3) Larsemann Hills; 4) Amery Oasis; 5) Drygalski Ice Tongue; 6) Marguerite Bay; and 7) James Ross Island. (**b**) Schematic map of the Tarn Flat deglaciated area with the Lake-1 and Lake-2 locations in red and the other two lakes with mounds previously reported by Guglielmin *et al*.^33^ in yellow; (**c**) aerial photograph of the Tarn Flat area taken on 31 December 2015 (courtesy Nicoletta Cannone), with the eskers on the right; (**d**) photograph of Lake-1 taken on 10 November 2014, courtesy of Michele Dalle Fratte. The frost mound is marked by the letter (M); (**e**) photograph (view from the north) showing a close view of the frost mound (M); the letter (B) marks the drilling rod (the picture was taken by Emanuele Forte on 18 November 2014).

Only in correspondence with the deeper trough is a relevant structure clearly visible that has lateral slopes, which were imaged by GPR (horizon LM on Fig. 3) with a mean dip of 20–30°, although with large lateral variations. The surface of this structure has an irregular shape (Fig. 4b), as also testified by its outcropping at the lake’s surface (Fig. 1e).

Below that mound, brines have been clearly identified using GPR data because of the high signal absorption in brines, which is due to their electrical conductivity, causing an extremely high intrinsic attenuation (Fig. 3). Moreover, there, for the first time, where brines were identified by GPR, they were also reached and sampled by a borehole (Fig. 5).

It is probable that the brine extrusion was accomplished by contemporary sediment and algae fragments as well as decimetric rock blocks, which blew up when they sometimes reached the actual mound surface (Fig. 1e). All such materials could be responsible for the high amplitude diffraction hyperbolas imaged on GPR data within the mound zone (Fig. 3).

Inside the mound, the GPR profiles imaged five different noncontinuous reflectors (labelled H0 to H4) that correspond to the sediment layers found in the borehole (Fig. 5). In detail, horizons H0 and H1 are both thin layers of sediments at a depth of approximately 1.5 and 2 m, and they are apparent in almost the entirety of the lake and also outside the mound zone, whereas H2 on the GPR sections, which most likely corresponds to the 2 cm thick silt/clay layer with organic material, is located at 308 cm in the core (Fig. 5a). It was imaged, although the reflection amplitude was very low across the mound on several profiles (see, e.g., Fig. 3). In any event, it is not continuous but shows abrupt lateral interruptions that could be responsible for the brine up-flow. On the other hand, the presence of organic material and fine sediments could explain the fact that only this horizon is also visible in a very high attenuating zone due to the higher electromagnetic contrast with the surrounding materials.

The thicker sediment layers H3 and H4 found in the borehole between 4.94 and 5.68 m were not imaged by the GPR just below the frost mound due to the extremely high attenuation of the electromagnetic waves that was most likely produced by the brines. In any event, such layers were interpreted on all the GPR profiles down to a depth of approximately 5 m below the lake surface (Figs 3 and 4).

Moreover, the morphology of the sedimentary basal level (H4), which is characterized by a marked deepening as obtained by the 3D data integration shown in Fig. 4, suggests that some rocky and sedimentary materials from the lake’s bottom were extruded towards the shallowest portion of the lake and that some of them reached the surface (Fig. 5).

The 3D reconstruction obtained by integrating the top of the bedrock (BD) and the top of the sedimentary layer H3 is provided in Fig. 4a. We used layer H3 instead of H4 because the depth of the former can be better defined on the GPR sections. Where the bedrock is directly in contact with the ice (i.e., around the borders of the lake), the surface shows an irregular morphology, whereas horizon H3 (as well as H4) is characterized by a smoother shape. The contact between BD and H3 is highlighted by the white dotted line in Fig. 4c, and the approximated extension of the brines is marked on the same figure by a black dotted line.

### Borehole stratigraphy

Based on a visual observation of the amount and distribution patterns of the included bubbles and/or sediment, the borehole presents different types of ice. The two more common ice types can be summarized as type I, which contains a certain amount of fine sediments (clay/silt) that are randomly distributed and bubbles that are in the main not elongated, and type II, which is constituted by alternating ice I and pure ice with primarily horizontally elongated bubbles.

The borehole stratigraphy (Fig. 5) is quite complex; the first 2.5 m is characterized by alternating ice I and II, whereas between 2.5 and 3.61 m the two types of ice alternate with thin levels of partially frozen brines.

At different depths, thin (1–2 cm thick) layers of sediments are present; they are silty-sand at the surface, at 1.51 and at 1.86 m (H0 in Fig. 5a) and silty/clay with some organic material at 3.08 m of depth (H2 in Fig. 5a), whereas a thicker (7 cm) layer of ice rich in debris (clasts > 2 mm) was further found at a depth of 204 cm (H1 in Fig. 5a).

Between depths of 3.61 m and 3.78 m, there is a 17 cm layer of ice with bubbles that is elongated perpendicular to the surface, followed by a pocket of liquid brine (B1) with an important flow until a depth of 3.98 m, where a 12 cm layer of ice with some organic material inclusions occurs. The thickest (0.84 m) pocket of liquid and flowing brines (B2) lies below.

Finally, between 4.94 m and the bottom of the borehole (5.68 m), there are two layers of frozen sediments (H3 and H4 in Fig. 5a) and one layer of pure ice between them. In detail, from 4.94 to 5.21 m, there is a layer of fine laminated sediments that is followed by a pure ice layer down to 5.53 m, where a 15 cm thick layer comprised of fine sediments that are very rich in organic material was found.

The salinity profile within the borehole (Fig. 5b) shows very low values of salinity (lower than 4 psu) until 2.5 m, where in correspondence with the alternating ice and the thin layers of partially frozen brines (B0), there are intermediate values (ranging between 10 and 28 psu), until brine B1. Below, the salinity of the ice layers is quite low, except for the deepest layer of pure ice, which showed a high value (53 psu).

The two pockets of liquid brines located between 3.78 and 3.98 m and between 4.10 and 4.94 m showed salinities that ranged between 84 and 92 psu in B1 and 74–75 psu in B2 (Fig. 5b). Those brines also had different pHs, which were equal to 7.22 for B1 and to 6.74 for B2.

To date the beginning of the sedimentation, the organic material contained in the deepest part of the H4 layer, bulk sediment was dated with AMS ^14^C, which gave a conventional age of 10410 ± 30 BP that corresponds to a calibrated age between 12406 and 12038 yr cal BP.

## Discussion

The shape of the lake troughs reflects the main flow direction of the Reeves glacier, as testified by several striae (Fig. 1c,d) and by the esker (Fig. 1c) not very far from Lake-1 and Lake-2, although it is not possible to exclude a possible structural control of those lineaments. The mound that is evidently highlighted above the main and deeper trough of Lake-1 is a frost mound that is clearly related to the extrusion of the brines imaged within the mound itself by the GPR survey and that was subsequently sampled.

Similar mounds of smaller extension and close to the Tarn Flat area were previously described by French and Guglielmin^34^ and Guglielmin *et al*.^33^. For the first time, we investigated and characterized such structures and determined that I) brines occur within the ice in the upper part and as pure liquid brines in the deeper portion and that II) several discontinuous sediment layers and randomly distributed blocks occur within the structure.

Both of those characteristics suggest that the mound should be related to multiple events of brine extrusions from a deeper reservoir, as testified by both the lateral interruptions of sediment layers H0, H1 and H2 and by their deformations. If that is the case, the source of the pressures that caused the ice heave must be related to peculiar hydrologic conditions. A hydraulic mechanism seems to be more plausible because it requires that water is injected under pressure from an underground hydrologic connection (talik) in an open system. A partial validation of such a hypothesis is the sensible pressure observed in the borehole, where an uplift of brine level B2 was observed with a flow rate that decreased with time (average of 9.6 l/hour). Such brines reached the lake surface approximately 40 hours after the borehole was drilled. It is certain that the pressure was much stronger in the past because remnants of brine with fragments of silty-sandy layers of sediments and algae are actually on the surface of the mound. Moreover, the fact that the salinity of the deeper brines (B2) is lower than that of the upper ones supports the hypothesis of a hydraulic open system because recharge from outside the lake basin can decrease the salinity that should otherwise increase in a closed system for repeated basal freezing events.

The only pressurized brine system confined beneath ice and sediment layers similar to the case herein described has been recently found and reported at Lake Vida^14^. At Lake Vida, the brine system was found at a depth of 16 m within the ice cover, and the brine rose to 10.5 m flowing within fractures and small channels, whereas at Tarn Flat we have similar brines that are confined (but not pressurized) in fractures only between 2.50 and 3.61 m (B0), and below those depths B1 and B2 are two different aquifers of pressurized liquid brines.

The extension of B1 and B2 is limited within the deeper trough (black dotted line in Fig. 5c), where pingo-like features (PLF) occur. Frost mounds or PLF are quite common at high latitudes in the Arctic^35^, but they are almost unknown in Antarctica, and in both cases brines were never found. Frost mounds were observed on the floor of the Arctic Ocean^36^ and also on Mars^6^. Mounds that appear as PLF on Mars were also described as surface reservoirs of solid carbon dioxide by Malin *et al*.^37^, and similarly the PLF found in the offshore permafrost of the Kara Sea in the Russian arctic by Serov *et al*.^38^ could lead to potential methanogenic blowouts in thawing permafrost. This event is not so remote in our case because gas bubbling was observed during the drilling operation, and gas sampling is planned for the next Antarctic summer.

Analysing both GPR profiles that did not cross the mound (e.g. Fig. 6b) or acquired on Lake-2 (Fig. 6c), we did not detect any brine, but in both cases a continuous highly reflective sedimentary layer was imaged. In Lake-2 it is almost horizontal, but in Lake-1 it shows a much more complex geometry.

From the geophysical point of view, the brines do not show precise upper limits because in this case we should find a clear strong reflector that marks this boundary. Conversely, the presence of brines was inferred by the lack of a GPR signal, which is strongly attenuated by the high electrical conductivity of the brines. The highly reflective horizons H3 and H4, which can be detected over a wide area of the lake (white dashed line in Fig. 4c), are most likely interpreted as the two thick horizons found in the deepest part of the core; they have a very large reflection coefficient, but some energy can still penetrate below as testified by the reflection from the top of the bedrock (marked BD in Fig. 3). This result is similar to that obtained in Lake Vida, where a continuous highly reflective horizon that was seen on a GPR survey and at first interpreted as the top of a brine body^11^ was recently re-interpreted with the help of new drillings as a sediment layer^14^. The peculiar “mirror-image” multiples produced below the brine reflectors and reported by Nobes *et al*.^39^ in GPR data close to Scott Base are not present in our dataset, although a “normal” multiple reflection produced by horizons H3 and H4 is easily recognized (Figs 3 and 6). GPR data clearly imaged and correlated with five distinct sedimentary horizons within Lake-1; some of them are more continuous (e.g., H2, H3 and H4), whereas others (HO and H1) are more irregular (Fig. 3). The effectiveness of the geophysical outcomes was demonstrated by the borehole validation both in terms of the sedimentary layers and brine reservoirs that were found. The GPR was further important in locating the borehole in the deepest part of the lake, where the brines were expected, and in evaluating the lateral continuity of the sediments.

The occurrence of the more surficial layers of sediment (H0, H1 and H2) within the ice both in the PLF and in the rest of the lake could have a similar origin to those found in Lake Vida, according to Dugan *et al*.^14^. Such layers could be related to surface runoff processes that accumulate sediments at the surface. If this hypothesis is correct, the sequence of ice and sediment layers at Tarn Flat may also record past hydrological changes, although those changes could not have been cyclic but oppositely show large discontinuities in the ice cover during prolonged cold/dry periods.

The deeper sediment layers H3 and H4 suggest a different origin, as proposed in Fig. 7a; in fact, they are limited only to the deeper part of the lake and partially directly lie on bedrock. These characteristics together with their finer grain sizes, higher organic contents and greater thicknesses suggest an original lacustrine deposition. After that period, during which the lake probably could have been completely or partially melted during the summer, as happens now in other lakes of the Tarn Flat at lower altitudes, the lake experienced a period when several episodes of surface runoff processes producing the accumulation of sediment layers (H2, H1 and H0). Only in a more recent and unknown time(s) the brines from outside the basin succeeded to blew up, breaking the overlying ice and sediment layers and forming the mound on the surface. From the palaeoclimatic point of view, the age of layer H4 obtained by the bulk sediment suggests that in this small lake, which is south of 70°S and perennially frozen (it was frozen even in the exceptionally hot summer of 2001/02, which was directly observed by one of the authors), the sedimentation should not have started prior to 12.4 kyr cal BP, i.e., approximately in the same period when sedimentation also occurred at Amery Oasis^22^ in the Northern Prince Charles Mountains. If confirmed by future drillings, the deglaciation age of this area will fit previous geomorphological reconstructions^28^,^40^ that hypothesized, although without any absolute ages, that the last glaciation in this area occurred at approximately 24–27 kyr BP.

Recently, Anderson *et al*.^41^ suggested that the ice-sheet was still grounded in this sector of Victoria Land at approximately 12 kyr BP, and the deglaciation of the area should therefore have occurred probably in the Early Holocene or at the end of the Pleistocene.

Considering that there is no evidence for higher levels of Lake-1, this small basin could preserve at least all the Holocenic hydrological and climatic changes although within a more limited thickness, for instance Lake Vida, where 8600 years are recorded in 27 m of ice and sediments; in other words, the sedimentary sequence of Lake-1 could be a more condensed chronological series.

Regarding the age of the PLF and therefore the age of the possible brine flows, we can only hypothesize that this event postdates the sedimentation of layer H0 because all the sediment layers within the ice are truncated by the slope of the PLF, but we cannot exclude that this feature was formed by successive episodes that cut the different sediment layers at different times.

## Methods

### Field data acquisition

We used a ProEx (Dual Channel configuration) GPR produced by Malå Geosciences that was triggered by an electromechanic odometer and equipped with two shielded antenna pairs with central frequencies equal to 500 and 1600 MHz. In the present work, we will only show examples taken from the 500 MHz dataset. A Trimble GPS that recorded a fix every 10 seconds was used for the absolute data positioning. The mean trace interval equalled 15 cm.

**Figure 2:**
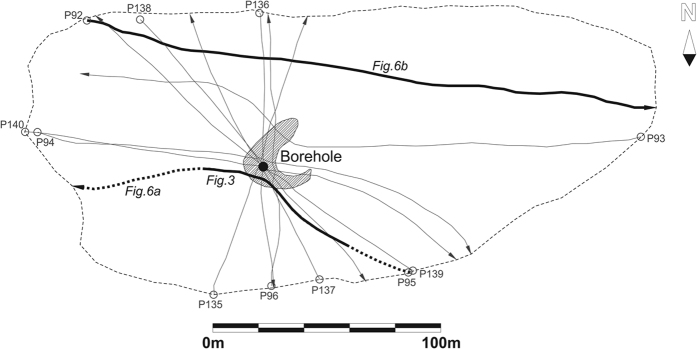
Lake-1 perimeter with the locations of the GPR profiles superimposed. The dashed area marks the zone where the frost mound is visible at the surface, and the borehole location is also shown.

In addition, a 51 mm diameter borehole that reached a depth of 5.68 m was drilled in the centre of the frost mound using a semi-portable core auger (Figs 1e and 2), exploiting the preliminary GPR results. The extracted core was stored at −20 °C at MZS prior to its delivery to the Beta Analytic laboratories (US) for ^14^C dating. During the coring, when we reached the first pocket of brine at 378 cm we interrupted the coring and collected brine in aquifer B1 using a peristaltic pump and sterile tubing. After collecting 5 litres of brine B1, we continued the core sampling of the following 12 cm and then re-inserted a new sterile tube to collect the brine from aquifer B2, which was located between 410 cm and 494 cm of depth. Finally, after collecting brine B2, we completed the core, reaching a depth of 568 cm.

### Data processing and laboratory analyses

We applied a processing flow to GPR data that included drift removal (zero-time correction), background removal, geometrical spreading correction and exponential recovery, bandpass filtering, 2D migration (f-k Stolt algorithm), and depth conversion. For the last two steps, we used a constant velocity value of 0.17 m/ns, considering both the reference value for pure ice and the results of dedicated diffraction hyperbola analyses, which all produced values very close to the previous one.

**Figure 3:**
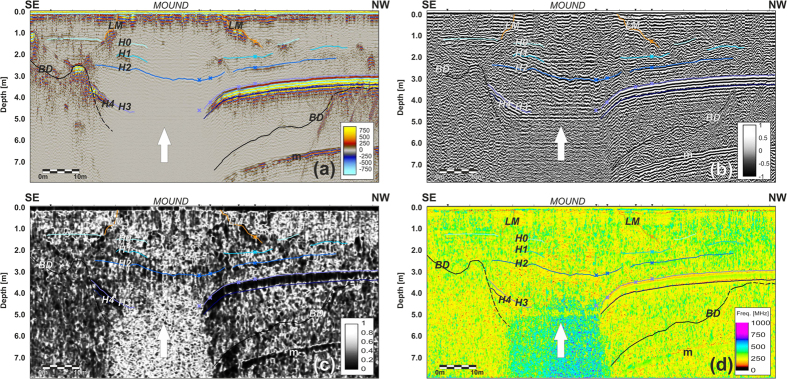
GPR Profile P95 carried out using the 500 MHz antenna processed (not migrated) and interpreted: (**a**) reflection amplitude; (**b**) cosine of the instantaneous phase; (**c**) *chaos* texture attribute; (**d**) dominant frequency. Horizons H0 to H4 have been interpreted as sediment layers; (BD) top of the bedrock; (LM) lateral limits of the frost mound; (m) multiple reflections. The white arrows schematically indicate the brine upwelling.

**Figure 4:**
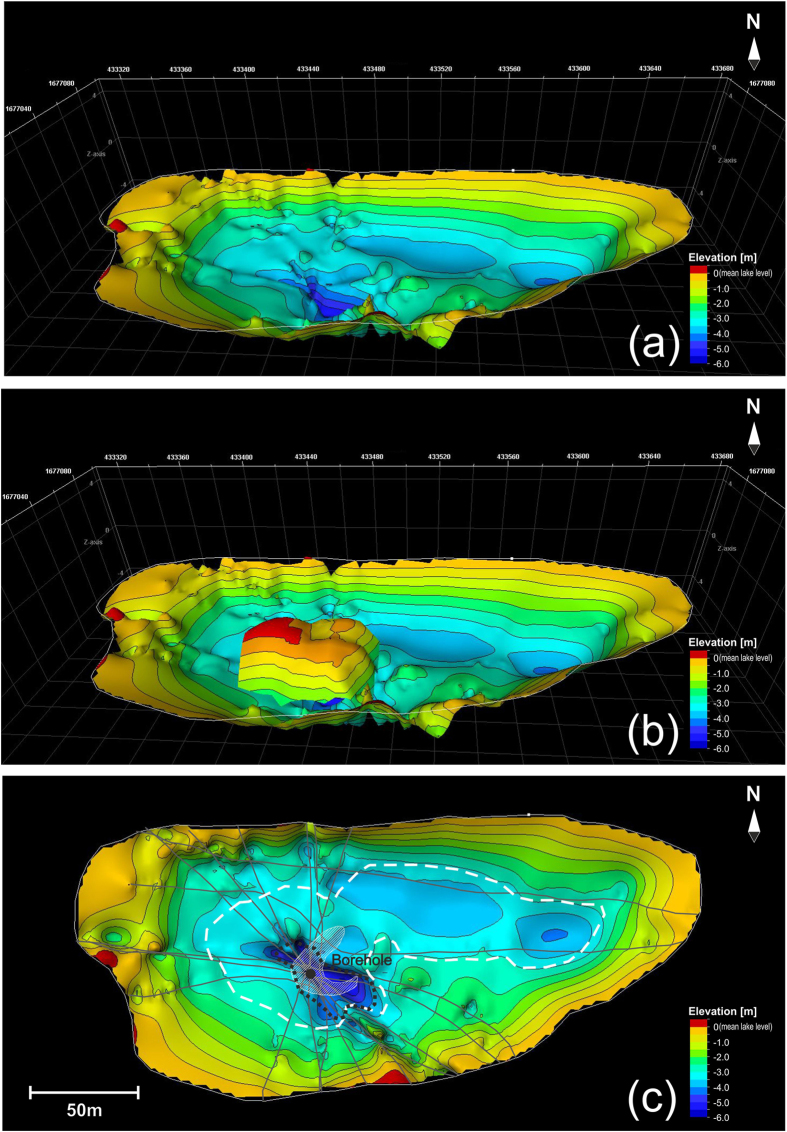
(**a**) 3D reconstruction of the combination of the top of the bedrock (BD) with the top of horizon H3; (**b**) same surface as in (**a**) with the flanks of the frost mound superimposed; (**c**) plain view of the same surface as in (**a**). The white dashed line marks the contact between the bedrock (outside) and the top of horizon H3 (inside), whereas the black dotted line indicates the approximated limit of the brines as obtained by the 3D GPR data integration.

**Figure 5:**
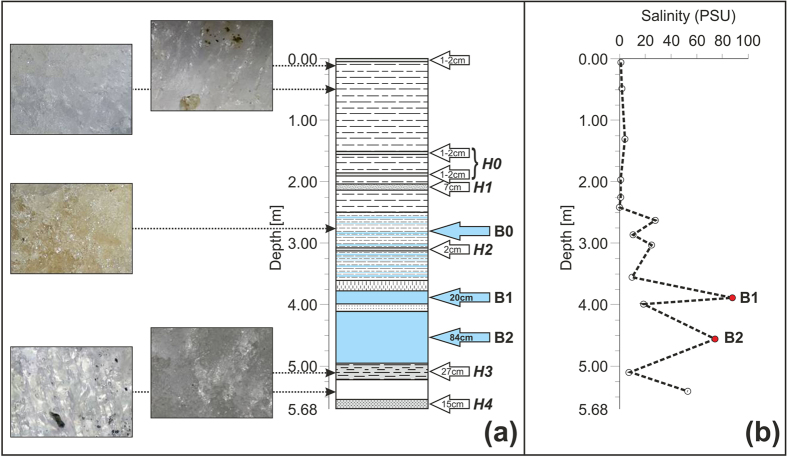
(**a**) Schematic stratigraphy of the borehole and photographs showing different types of sampled ices. The grey levels represent different types of sediments, the white levels depict ice layers, and the light blue levels are the zones with brines (B0, B1 and B2); (**b**) salinity log of samples taken from the borehole core: B1 and B2 indicate the brine levels. See the text for further details.

**Figure 6:**
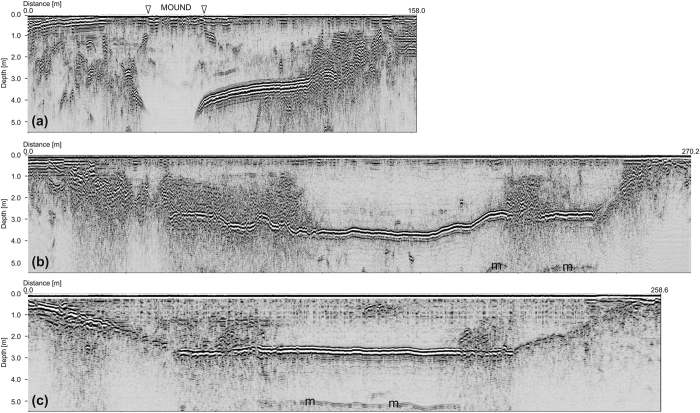
Examples of different migrated and depth converted GPR profiles of Lake-1 (**a**,**b**) and Lake-2 (**c**). (**a**) Profile P95 crossing the frost mound; (**b**) profile P92 located to the north of the mound and not crossing it; (**c**) profile acquired on Lake-2. The peculiar effect of the mound and the brines is apparent on (**a**), whereas the profiles in (**b**,**c**) are quite similar, even if the one acquired on Lake-2 seems to image a simpler situation with an almost flat sedimentary layer, regular bedrock flanks on both sites, and quite homogeneous ice without clear sedimentary layers or diffractive bodies. Multiple reflections are marked with an (m).

**Figure 7:**
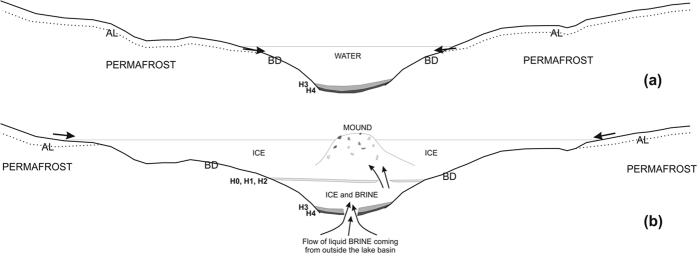
Scheme of the evolution of Lake-1 over time and a possible explanation of the PLF development. (**a**) The beginning of sedimentation on the lake bottom did not occur prior to 12 kyr cal BP, as documented by the ^14^C dating of the H4 layer. The lake could be melted during the summer in a probable wetter climate; (**b**) formation of the PLF after the deposition of lake sediments H4 and H3 and after the deposition of the other thin layers of sediments related to surface runoff processes. The age of the brine extrusion responsible of the formation of the PLF and of the breaking of the different sediments layers is unknown but is reasonably within the Holocene. Legend: AL = active layer; BD = bedrock.

For a better and more constrained GPR data interpretation, we calculated GPR attributes that were then integrated and correlated (Fig. 3). Such a technique has been used with reflection seismic data since approximately 40 years ago^42^,^43^ but has not yet seen widespread application to GPR analysis. Recently, attributes were calculated on GPR data to obtain an improved characterization of the frozen materials within a glacieret^44^. In the present work, among the “classical” instantaneous attributes, we also calculated the textural ones (Fig. 3c). Textural analysis was first applied to image processing^45^ and then became a popular tool in reflection seismic data interpretation to better highlight complex stratigraphic features. Due to the intrinsic high resolution of GPR signals, textural attributes are expected to be very powerful for emphasizing both the general signature of an investigated area and details that are difficult to image with standard analysis techniques^46^. In the present case, the phase-related attributes allowed a more constrained interpretation of low amplitude reflectors (e.g., H2 in Fig. 3b) and texture highlighted the most relevant subsurface structures, emphasizing the peculiar signatures of different materials (Fig. 3c), whereas frequency-related attributes discriminated domains with specific spectral characteristics, in particular providing a better imaging of the zone with the brines (Fig. 3d).

The salinities and pHs of the brines were measured at MZS and at Messina CNR laboratories using refractive index and a potentiometric method, respectively.

The ^14^C dating samples were sealed in polyethylene bags and frozen at −20 °C. They were kept frozen until they were sent to the Beta Analytic laboratories (Miami, US) for radiocarbon dating. There the frozen sediments were subjected to acid washes following Beta Analytic standard procedures. After pre-treatment, the samples for radiocarbon dating were prepared for AMS by converting them into graphite. The calibrated ages were calculated with the OxCal 4.2 software program^47^ using the SHCal13 14C Southern Hemisphere atmosphere dataset^48^. The radiocarbon age datum is reported as conventional radiocarbon years BP (^14^C yr BP) ± σ and as calibrated age ranges with a 2σ error (95.4%) (cal. yr BP; relative to AD 1950).

## Additional Information

**How to cite this article**: Forte, E. *et al*. Pressurized brines in continental Antarctica as a possible analogue of Mars. *Sci. Rep.* **6**, 33158; doi: 10.1038/srep33158 (2016).
